# Dominance induction of fruitlet shedding in *Malus × domestica *(L. Borkh): molecular changes associated with polar auxin transport

**DOI:** 10.1186/1471-2229-9-139

**Published:** 2009-11-26

**Authors:** Valeriano Dal Cin, Riccardo Velasco, Angelo Ramina

**Affiliations:** 1 Department of Environmental Agronomy and Crop Science, University of Padova, Viale dell'Università 16, 35020 Legnaro (Padova), Italy; 2 Experimental Institute for Agriculture, via Mach 2 San Michele all'Adige, 38010 Trento, Italy; 3 Horticultural Sciences, University of Florida, Gainesville, PO Box 116090, USA

## Abstract

**Background:**

Apple fruitlet abscission is induced by dominance, a process in which hormones such as auxin, cytokinins and strigolactone play a pivotal role. The response to these hormones is controlled by transcription regulators such as Aux/IAA and ARR, whereas auxin transport is controlled by influx and efflux carriers.

**Results:**

Seven partial clones encoding auxin efflux carriers (*MdPIN1_A*, *MdPIN1_B*, *MdPIN10_A*, *MdPIN10_B*, *MdPIN4*, *MdPIN7_A *and *MdPIN7_B*), three encoding auxin influx carriers (*MdLAX1*, *MdLAX2 *and *MdLAX3*) and three encoding type A ARR cytokinin response regulators (*MdARR3*, *MdARR4 *and *MdARR6*) were isolated by the use of degenerate primers. The organization of the PIN multigene family in apple is closer to *Medicago truncatula *than to *Arabidopsis thaliana*. The genes are differentially expressed in diverse plant organs and at different developmental stages. *MdPIN1 *and *MdPIN7 *are largely more expressed than *MdPIN10 *and *MdPIN4*. During abscission, the transcription of these genes increased in the cortex whereas in the seed a sharp fall was observed. The expression of these genes was found to be at least partially controlled by ethylene and auxin.

**Conclusion:**

The ethylene burst preceding abscission of fruitlets may be responsible for the decrease in transcript level of *MDPIN1*, *MDARR5 *and *MDIAA3 *in seed. This situation modulates the status of the fruitlet and its fate by hampering the PAT from the seeds down through the abscission zone (AZ) and this brings about the shedding of the fruitlet.

## Background

Abscission is a coordinated process tightly regulated by the interplay of several factors, among which auxin and ethylene play a pivotal role [1,2]. Leaf deblading and ethylene application lead to premature abscission of the organ due to the disruption of the auxin flux and activation of the abscission zone (AZ) at the base of the petiole [3,4].

In the commonly accepted model, as demonstrated in the Arabidopsis *etr1-1*, ethylene coordinates abscission. In this mutant, flower abscission is significantly delayed because the ethylene receptor (ETR1) is hampered in ethylene binding activity, leading to partial ethylene insensitivity. Although ethylene accelerates abscission, it is strictly not necessary for shedding, indicating that a very complex interplay of events control the process [5-7]. Indeed, it has been shown that ethylene-dependent and -independent pathways converge in determining flower abscission [8].

It has also been postulated that prevention of abscission requires a continuous and constant auxin transport through the AZ [1]. Besides preventing abscission, auxin regulates a tremendous number of processes, for instance root meristem activity, organogenesis, and vascular tissue differentiation [9-11]. Only recently the outstanding complex mode of action of auxin has been partially unraveled [12]. The most common auxin in plant, indol-3-acetic acid (IAA), binds and is perceived by TIR1, an F-box protein [13]. TIR1 interacts in the SCF complex to bring about the degradation of Aux/IAA transcriptional regulators [14]. These proteins are active repressors of auxin responsive genes and are encoded by a large multigene family [15]. Auxin applications enhance the transcript amount of most of the Aux/IAAs in several species [16-19]. Another enthralling field concerns auxin transport [20], which can be classified as either polar (PAT) or non polar. However, the PAT is acquiring ever-growing interest and may be the most important means of auxin relocation [21]. IAA is taken up into the cell by a combination of lipophilic diffusion, symport via AUX and LAX (LIKE-AUX1) permeases, and ATP-dependent transport by a P-glycoprotein [22-25]. Auxin export is mediated by PIN-FORMED (PIN) facilitators and by ATP activated PGPs (Phosphoglycoproteins) [26-30]. PINs and PGPs were shown to characterize coordinated and independent auxin transport mechanisms, and function interactively in a tissue-specific manner [31]. Nevertheless, the function of the PGPs is non-specific and mainly applies to auxin excess [32]. As a matter of fact, it is the asymmetric cellular localization of PIN proteins that determines the direction of the auxin flow [20]. Although different PINs are implicated in specific developmental processes, there seems to be redundancy as indicated by the ectopic expression of PIN proteins in some mutant combinations [20,33,34].

The modes of action of auxin and ethylene elucidated in *A. thaliana *have been extended to other model species such as tomato [35,36]. Yet, little is known about the interactions between these two hormones during abscission induction of organs other than debladed leaves or senescing flowers. In particular, the apple cluster during the immature fruit drop represents an ideal system to study the shedding of actively growing organs [37]. At this developmental stage, the shedding process involves almost exclusively lateral fruitlets in which abscission is preceded by an increase in ethylene biosynthesis and sensitivity [38-40]. According to the correlative basis reknown model the central fruitlet exerts a dominant effect over lateral fruitlets because it is at a more advanced stage of development [37,41]. As assessed by the canalization theory the strong auxin flow coming from the central fruitlet, down to the peduncle through the AZ into the twig, depolarizes the weak auxin flows from the lateral fruitlets causing their abscission [42].

Apical dominance is a complex physiological process largely controlled by auxin and its interaction with two additional hormones: cytokinins and MAX (more axillary branching [43-45]. Cytokinins produced in the roots are directed to organs (shoot apical meristems, fruits, etc) whose sink strength is related to their ability in producing and exporting auxin [46]. This process directs more cytokinins which stimulate growth [47,48]. In the case of apical dominance of shoot meristems, lateral bud outgrowth occurs when the auxin flow from the apex is hampered, dominance is weakened, and cytokinins are redirected to axillary meristems [43,44]. Besides the main cytokinin stream coming from the roots, the hormone can also be produced in other tissues. For instance, following decapitation, a prompt increase in transcripts for the key enzyme in cytokinin biosynthesis, adenosine phosphate-isopentenyltransferase, occurs in the stem xylem [49]. The cytokinins produced here may then be translocated into the axillary meristems where they stimulate the lateral bud outgrowth. The cytokinin signaling relies on a two-component signal transduction system and upon activation it determines changes in transcript level of genes encoding proteins, such as the same cytokinin response regulators (ARRs type A) which are involved in various processes [50,51].

The second hormone which interacts with auxin is MAX, a carotenoid derived compound which has been only recently characterized as strigolactone, but its existence had been proven before by the discovery of the pea *rms*, petunia *dad *and *A. thaliana max *mutants [52-55]. These plants display excessive branching, indicating that strigolactone is a positive regulator of apical dominance. Among the several genes related to this hormone, *AtMAX2 *encodes an F-box protein [55]. Although the F-box protein is not required for the synthesis of MAX it is involved in the transduction of the signal at the level of the node [56,57].

The isolation, characterization and expression of some genes encoding elements involved in auxin transport, as well as of type A *MdARRs *and *MdMAX2*, were pursued to elucidate at the molecular level the interactions among ethylene, PAT and cytokinin in relation to the immature apple fruit abscission,

## Results

### Identification of elements involved in PAT in *MalusXdomestica*

**Figure 1:**
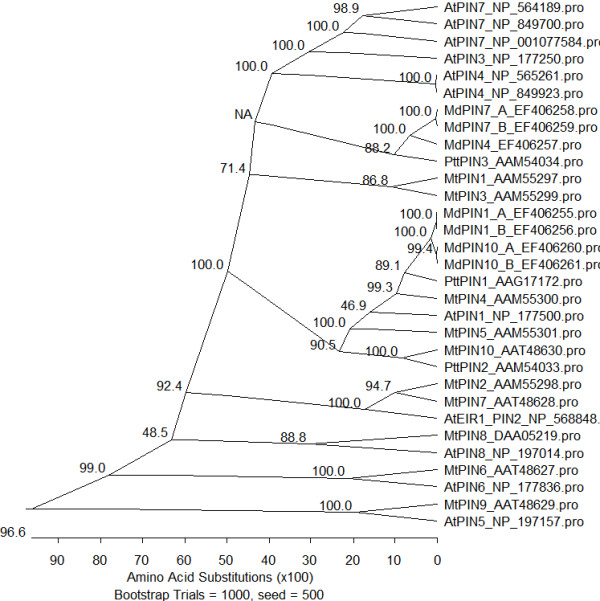
**PIN Phylogenetic tree**. Phylogenetic study of the PIN isolated in this study (*Malus × domestica *(*Md*)) with those of *Arabidopsis thaliana *(At), *Medicago truncatula *(Mt), *and Populus tremula × Populus tremuloides *(Ptt). (*MdPIN1_A*, EF406255; *MdPIN1_B*, EF406256; *MdPIN4*, EF406257; *MdPIN7_A*, EF406258; *MdPIN7_B*, EF406259; *MdPIN10_A*, EF406260; *MdPIN10_B*, EF406261; *AtPIN1*, NP_177500; AF089085; *AtPIN2*, NP_568848; *AtPIN3*, NP_177250; *AtPIN4*, NP_565261, NP_849923; *AtPIN5*, NP_197157; *AtPIN6*, NP_177836; *AtPIN7*, NP_564189, NP_849700, NP_001077584; *AtPIN8*, NP_197014; *MtPIN1*, AAM55297; *MtPIN2*, AAM55298; *MtPIN3*, AAM55299; *MtPIN4*, AAM55300; *MtPIN5*, AAM55301; *MtPIN6*, AAT48627; *MtPIN7*, AAT48628; *MtPIN8*, DAA05219; *MtPIN9*, AAT48629; *MtPIN10*, AAT48630; *PttPIN1*, AAG17172; *PttPIN2*, AAM54033; *PttPIN3*, AAM54034). Bootstrap values are reported.

**Figure 2:**
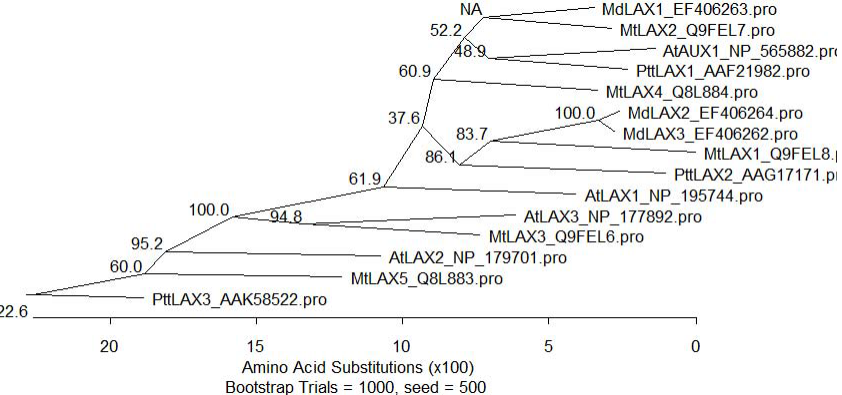
**AUX Phylogenetic tree**. Phylogenetic study of the AUX and AUX-Like isolated in this study (*Malus × domestica *(*Md*)) with those of *Arabidopsis thaliana *(At), *Medicago truncatula *(Mt), *and Populus tremula × Populus tremuloides *(Ptt). (*MdLAX1*, EF406263; *MdLAX2*, EF406264; *MdLAX3*, EF406262; *AtAUX1*, NP_565882; *AtLAX1*, NP_195744, NP_974719; *AtLAX2*, NP_179701; *AtLAX3*, NP_177892; *MtLAX1*, Q9FEL8; *MtLAX2*, Q9FEL7; *MtLAX3*, Q9FEL6; *MtLAX4*, Q8L884; *MtLAX5*, Q8L883; *PttLAX1*, AAF21982; *PttLAX2*, AAG17171; PttLAX3, AAK58522). Bootstrap values are reported.

During a differential display study between abscising and not abscising fruitlet populations, several clones related to auxin were isolated. However, only two transporters were found: with one encoding an auxin hydrogen symporter whereas the other one was too short and located in a highly conserved region which made further studies complicated [58]. We then pursued the isolation of other elements involved in auxin transport by the amplification of fruitlet cDNAs with degenerate primers and the following 3' race. This approach allowed the identification of seven *PIN *(Additional file 1) and three *LAX *(Additional file 2) partial clones. The name was chosen according to the highest level of similarity with the PIN of *A. thaliana *and *M. truncatula*, two dicotyledonous species whose members have all been well characterized at the genomic level. *MdPIN1_A *(EF406255) *MdPIN1_B *(EF406256), *MdPIN10_A *(EF406260), and *MdPIN10_B *(EF406261) are likely to be orthologous to *AtPIN1*, whereas *MdPIN7_A *(EF406258), *MdPIN7_B *(EF406259), and *MdPIN4 *(EF406257) may be orthologous to *AtPIN3*, *AtPIN4 *or *AtPIN7 *(Figure 1 and Additional file 3). Nevertheless, this association has to be definitely proven by functional and synteny studies. The isolation of different alleles (presented here with a letter) and the fact that *MdPIN1 *and *MdPIN10 *are different genes and not allelic forms was ascertained by the isolation and comparison of the genomic clones (*MdPIN1*: EF406268; *MdPIN10_A*: EF406269; *MdPIN10_B*: EF406270). The amplification of the genomic clones allowed the identification of the third, fourth and fifth intron as indicated in the work by [59]. The comparison of these regions indicated that the third intron was identical among the three clones whereas the fourth one was identical only between *MdPIN10_A *and *MdPIN10_B*. The fifth intron was the most divergent, but the identity between the two alleles of *MdPIN10 *was still 97.1% (Additional file 4). Concerning the *LAX *genes, *MdLAX1 *(EF406263) may be orthologous to *AtAUX1*, whereas *MdLAX2 *(EF406264) and *MdLAX3 *(EF406262) are more divergent and closer to *AtLAX *(Figure 2 and Additional file 5).

Since fruitlets are actively growing organs and auxins install apical dominance by directing the cytokinin stream derived from the root, some elements involved in cytokinin signal transduction were also isolated. These clones showed a high level of similarity to type A response regulators (ARRs, *A. thaliana *Response Regulators) of several species (results not shown) and were named according to the most closely related *AtARR*: *MdARR6 *(EF406267), *MdARR3 *(EF406265) and *MdARR4 *(EF406266).

### Expression analysis in the different tissues

**Figure 3:**
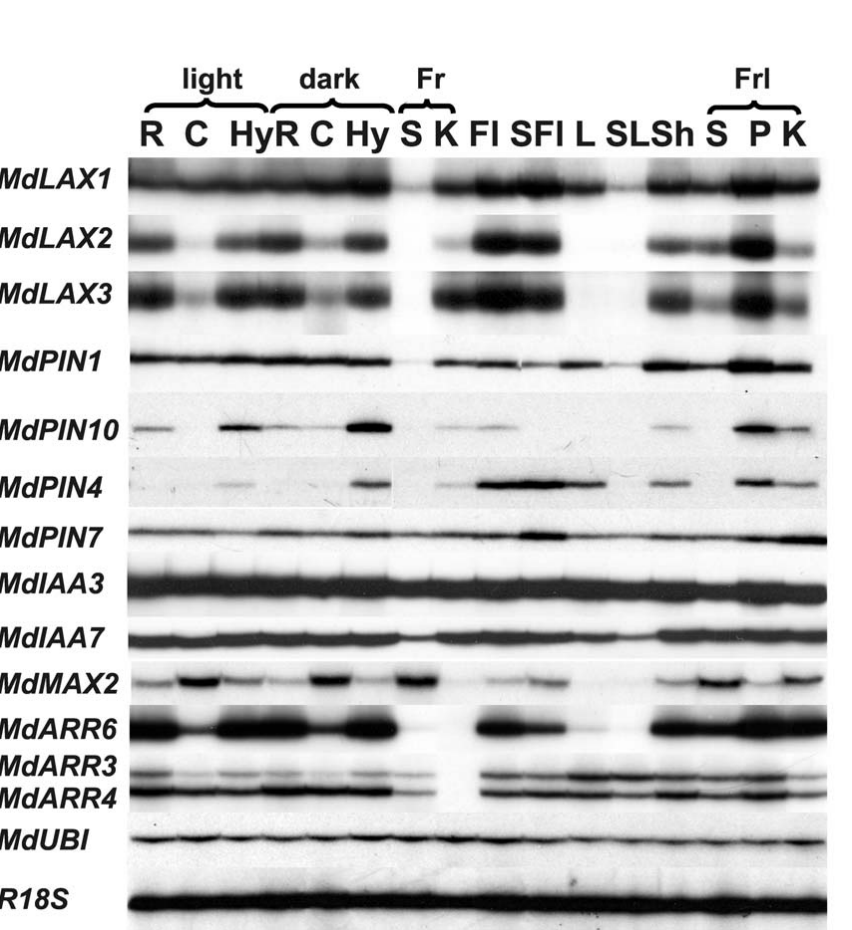
**Expression study in different organs of *MalusXdomestica***. Description: Expression analysis in different tissues and at different developmental stages performed by RT-PCR. Root (R), cotyledon (C) and hypocotyl (Hy) were collected from seedlings after 4 days of de-etiolation or left in the dark. Seed (S) and cortex (K) excised from fruit (Fr) at commercial harvest. Flowers (Fl) collected at full bloom and senescing un-pollinated flowers (SFl) were those not pollinated. Leaves were divided in mature fully expanded leaf (L), senescing leaf (SL) and shoot (leaflet and stem) (Sh). Fruitlets (Frl) were collected at 7 days APF and seed (S), peduncle (P) and cortex (K) dissected.

The RT- PCR expression analysis performed with ^33^P labeled degenerate primers visualized the relative level of expression among the different PIN genes. The high intensity of the band of *MdPIN1 *and *MdPIN7 *indicates that the transcripts of these genes are largely predominant on *MdPIN10 *and *MdPIN4 *(Additional file 6). This result was also confirmed by the number of cycles used in the RT-PCR: on average, *MdPIN1 *expression was studied at 33 cycles, *MdPIN7 *at 32 cycles, *MdPIN10 *at 35 cycles and *MdPIN4 *at 37 cycles (Table 1). Results showed a differential expression of the orthologs (Figure 3). *MdLAX1 *transcripts accumulated in all the tissues, although the signal was barely detectable in fruit seed and senescing leaf. *MdLAX2 *and *MdLAX3 *transcripts showed a similar pattern: in the seedlings the transcripts accumulated mainly in root and hypocotyl, whereas in fruit mainly in cortex. The transcripts were also abundant in the flower. Transcripts decreased during leaf ageing (from shoot to senescing leaf). Transcripts were also detected in shoot, whereas in fruitlet, the highest amount was observed in peduncle. *MdPIN1 *transcripts displayed a similar pattern to *MdLAX1*, with the signal slightly decreasing during flower and leaf senescence. *MdPIN10 *transcripts were mainly detected in seedlings grown in dark, above all in hypocotyl and peduncle; the transcripts slightly decreased along senescence of both flower and leaf. *MdPIN4 *displayed a pattern similar to *MdPIN10*, except in the flower where the signal was strong and apparently not affected by ageing. *MdPIN7 *transcripts were detected in all organs, with a slight increase in the seedlings grown in dark compared to those grown in light, and during flower senescence, whereas a decrease was observed in senescing leaf. *MdIAA3 *transcripts were present in all tissues at similar level, whereas *MdIAA7 *transcripts displayed a decrease during both flower and leaf senescence, and a weaker signal in seed than in peduncles and cortex. The strongest *MdMAX2 *signal was observed in cotyledons of light grown seedlings and in seeds of ripe and immature fruits. Furthermore, the transcripts accumulated preferentially in shoots rather than in fully expanded and senescing leaves. Besides fruit cortex and leaf, *MdARR6 *transcript accumulation pattern was the opposite to that of *MdMAX2*, with the signal decreasing in senescing flower and leaf. The *MdARR3 *signal, although weak in all tissues, was mainly detected in the root of light grown seedlings. In the other tissues, transcripts mainly accumulated in leaf. *MdARR4 *transcripts accumulated preferentially in dark grown seedling and, opposite to *MdARR3*, transcript amount decreased along leaf ageing.

**Table 1:** List of primers utilized in the expression analysis

Gene	Tm	**N**°**cycles**	Primer forward	Primer reverse	Accession number
*MdLAX1*	57	31	5'-AGTGCTATCAGTGCAAGC-3'	5'-CCGGAGAATTTTTCTGACTG-3'	EF406263

*MdLAX2*	*57*	31	5'-CTACCACCACCACATCAC-3'	5'-GGAAACACCACCAATTCAC-3'	EF406264

*MdLAX3*	*57*	31	5'-CATCACTAAGCTTCTCTCTC-3'	5'-CAACACCAATCCACTCTTC-3'	EF406262

*MdPIN1*	*71*	33	5'-ATTTTACCACGGCGGGCAAGG-3'	5'-CCTTCCCGCCATTGTTGTCCT-3'	EF406255/EF406256

*MdPIN10*	*71*	35	5'-CGGCGGACAGAATAATGCAGT-3'	5'-GAAGATCCTTCCCGCCATTGG-3'	EF406260/EF406261

*MdPIN4*	*71*	37	5'-ACAATTCAACAGCAGCAGCA-3'	5'-CGGTCCCACCAAAAACATGG-3'	EF406257

*MdPIN7*	*71*	32	5'-TTTGGGTTTTATCCCGCGCAGA-3'	5'-CGGCGGCTGCCTGATTTTTGC-3'	EF406258/EF406259

*MdIAA3*	*62*	29	5'-GGTTGAGAATCATGAAGGGT-3'	5'-AAAGCCCGAGCTCTATGTCT-3'	DQ848594

*MdIAA7*	*64*	30	5'-AATGCAAGAACAGGAGCTGA-3'	5'-TCCAATCAGTCGAACAACCT-3'	DQ848598

*MdAHS*	*58*	30	5'-CTTCGTTGGGTCAATTACG-3'	5'-GGCAATAATCACTCCAAGG-3'	EF406254

*MdMAX2*	*61*	30	5'-ACATTGCGGAAGCTCTTC-3'	5'-GCATCTTCGAATCGACAAC-3'	DY242015

*MdARR6*	*61*	30	5'-GCATCATCATCTCCCTCCTCT-3'	5'-CAATCCATCGTGTGCAATCTC-3'	EF406267

*MdARR3/4*	*61*	30	5'-TGATCCGACGATTGCTCCATC-3'	5'-CAGCGTTGGTCATTTTCAACC-3'	EF406265/EF406266

*MdUBI*	*61*	28	5-CATCCCCCCAGACCAGCAGA-3	5-ACCACGGAGACGCAACACCAA-3	DQ438989

*r18S*	*62*	25	5-GTTACTTTTAGGACTCCGCC-3	5-TTCCTTTAAGTTTCAGCCTTG-3	

Specific primers utilized for the expression analysis. Tm indicates the annealing temperature used in the PCR reaction. The number (N°) of cycles is that utilized in the experiments reported. The accession number is also reported in the right column.

### Expression analysis during abscission induction

**Figure 4:**
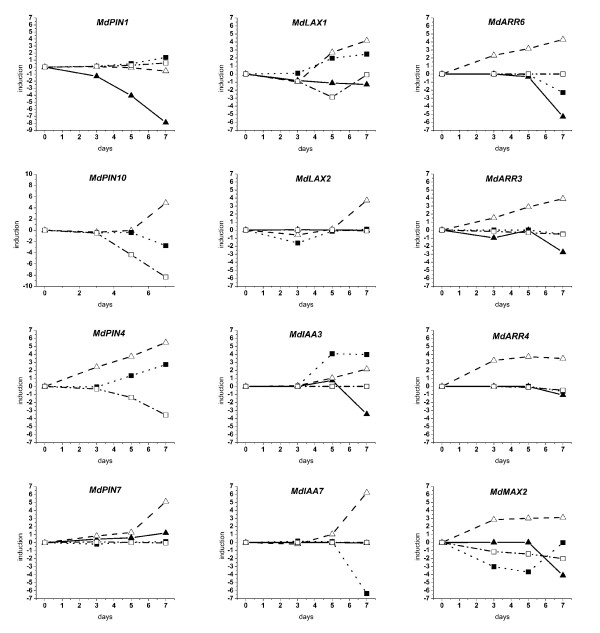
**Expression study during abscission induction**. Expression analysis in seed (solid triangle, continuous line), cortex (open triangle, dashed line), peduncle (solid square, dotted line) and AZ (open square, dashed-dotted line) performed by RT-PCR during abscission induction from 15 to 22 days APF. Samples were collected after triggering abscission with BA 15 days APF (time 0) and 3, 5 and 7 days later. Results are presented as folds induction compared to the T0 of the ratio between the expression level in the abscising fruitlets and in the persistent ones, corrected for the internal control (ubiquitin).

*MdPIN1 *transcript amount displayed a dramatic decrease in seeds whereas not much difference was observed in the other tissues (Figure 4). *MdPIN10 *transcripts increased in cortex by day 7 with a concurrent slight decrease in peduncle, whereas in AZ the decrease started already at day 5. *MdPIN4 *transcripts increased throughout the experiment in cortex, whereas in peduncle the increase was moderate and started later, concurrently with a decrease in AZ. *MdPIN7 *expression showed only a late increase in cortex. *MdLAX1 *transcripts slightly declined in seeds and increased in both cortex and peduncles. In AZ a transient decrease was observed at day 5. Since *MdLAX2 *and *MdLAX3 *showed the same pattern of expression, only results related to *MdLAX2 *are presented in Figure 2. Besides a slight transient decrease at day 3 in peduncle and a late surge in cortex, transcript levels remained unchanged along the experiment. *MdIAA3 *transcripts decreased by day 7 in seeds and increased by day 5 and 7 in peduncles and cortex, respectively. *MdIAA7 *transcript amount significantly increased in cortex at day 7 along with a dramatic decrease in peduncles. *MdARR6 *mRNA amount steadily increased in cortex, whereas in seeds and peduncles a decrease was observed at day 7. Analogously, *MdARR3 *transcripts increased in cortex and decreased in seeds and slightly in peduncles. Concerning *MdARR4*, mRNA amount already increased in cortex at day 3, then remained constant. *MdMAX2 *transcripts decreased late in seeds and increased at day 3 in cortex. The up-regulation was maintained in the following dates: in peduncle a transient decreased at day 3 and 5 was observed whereas in AZ mRNA gradually declined along the experiment.

### Peduncle development

**Figure 5:**
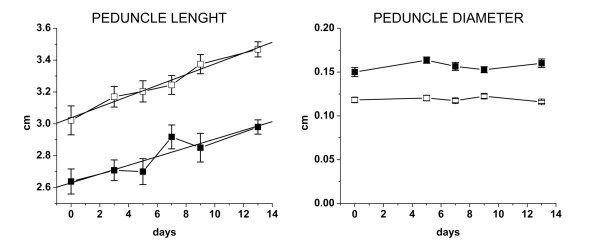
**Peduncle growth**. Peduncle length and diameter of the abscising fruitlets (open squares) and persistent fruitlets (solid squares) during abscission induction from 15 to 29 days APF. Samples were collected 3, 5, 7, 9 and 13 days after triggering abscission with BA at T0.

Peduncle growth was monitored along abscission up to 14 days after BA application (Figure 5). The length of the NAF peduncles was significantly shorter than the AF along the whole experiment. The initial length of the peduncles, according to the value of the intercepts calculated by a linear regression, was 2.63 cm and 3.04 cm for NAF and AF, respectively. On the other hand, according to the line equations (a regression value of 0.92 in NAF and 0.99 in AF), the growth rate of the fruitlet peduncle was similar with values of 0.027 and 0.034, respectively. Concerning diameter, the NAF was characterized by a thicker peduncle (0.15 cm) than the AF (0.12 cm), but no relevant changes were observed along the experiment.

### Auxin effect on PIN transcripts

Auxin application determined a dose-dependent response in PIN transcripts. Nevertheless, there was a clear increase only in *MdPIN4 *and *MdPIN10*, whereas in the case of *MdPIN1 *and *MdPIN7 *auxin application seemed only to counteract the natural decrease in transcripts as observed in the mock control (Additional file 7).

### Ethylene effect on transcript accumulation

**Figure 6:**
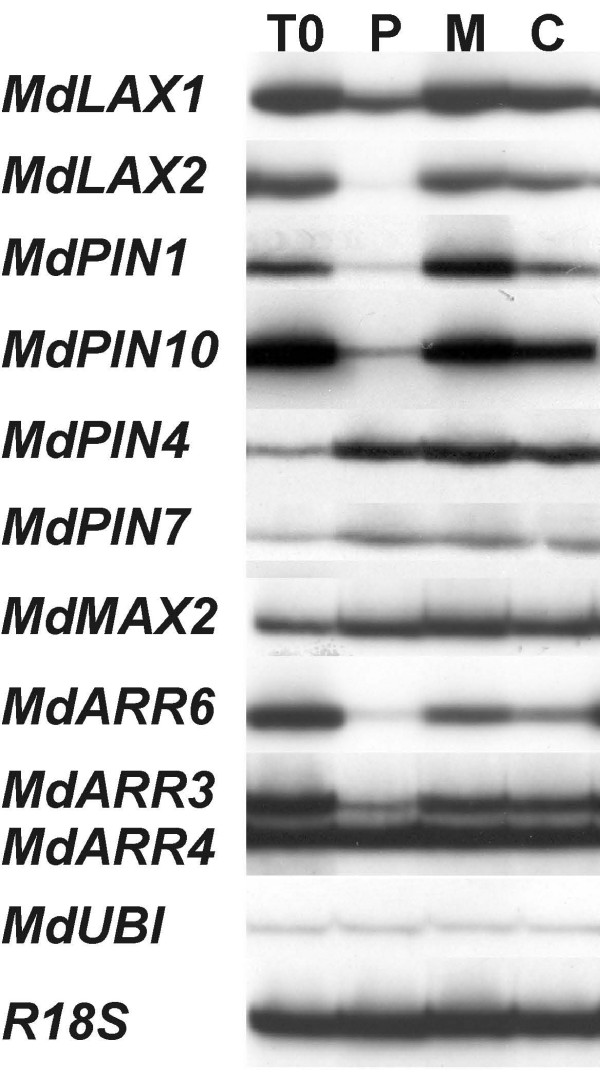
**Ethylene effect on transcript accumulation**. Expression analysis in peduncle of fruitlet clusters after 24 hours of propylene treatment (P) or 1-MCP (M) application. The control (C) represents untreated fruitlets after 12 hours from the beginning of the experiment (T0).

Fruitlet clusters were flushed with propylene or treated with 1-MCP for 24 hours and expression analysis was carried out in peduncles (Figure 6). Compared to the control, *MdLAX1*, *MdLAX2*, *MdPIN1*, *MdPIN10*, *MdARR6 *and *MdARR3 *transcript amount was decreased by propylene and increased by 1-MCP, especially in the case of *MdPIN1*. As far as *MdPIN4*, *MdPIN7 *and *MdMAX2 *are concerned, the transcripts accumulated throughout the experiment in an ethylene-independent manner, whereas no chemical effect was observed on *MdARR4 *transcripts.

## Discussion

### Which are the orthologs?

The isolation of various PIN and LAX clones in *Malus × domestica *indicates the presence of multigene families. Because of the identical expression profile and the high identity values, *MdLAX2 *and *MdLAX3 *are likely to be alleles. On the contrary, the alignment of the PINs presented here with the PINs of *A. thaliana *and *M. truncatula*, the isolation of the two genomic alleles of *MdPIN10 *and the differences at the level of the introns, and expression pattern of *MdPIN1 *and *MdPIN10 *indicate that they may be paralogous, as it has been reported for *MtPIN4 *and *MtPIN5 *[59]. The same organization was found in *Vitis vinifera*, *Populus trichocarpa *and *Oryza sativa*. Interestingly, *A. thaliana *presents a transcript variation of the PIN1 gene (Additional file 8). Another interesting point is that *MdPIN7 *and *MdPIN4 *are closely related to each other as found for *MtPIN1 *and *MtPIN3*, whereas in *A. thaliana*, besides *AtPIN3 *and *AtPIN7 *(showing a high level of similarity) a third member, *AtPIN4*, is present in the same cluster. The overall data indicate a different organization of the PIN members in the species discussed here. Similar organization for this cluster was found in *Populus *and *vitis *but not in *Oryza *(Additional file 8). This may be due to duplication and specialization of the members along plant speciation after *P. patens*. The organization of the auxin influx carriers was not completely conserved either (Additional file 9) This observation also highlights the difficulty in identifying the physiological orthologs of Arabidopsis genes.

### The expression of the PAT elements is driven by external and internal cues

During germination when a young plant has to grow through the soil particles and reach the surface, the cotyledons remain upright and the hypocotyl keeps on elongating until it reaches the light. The difference between hypocotyl length of seedlings grown in dark and seedlings during de-etiolation was also observed in our experiment (data not shown). Expression analysis performed on the seedlings indicated that light mainly affects transcript amount of the auxin efflux carriers (*MdPIN10 *and *MdPIN4*) in hypocotyl as reported in pea, whereas the influx carrier transcript quantity was barely changed (*MdLAX1*) [60]. According to the results, light negatively regulates PIN expression during germination. Considering the expression domains of the Arabidopsis PINs at this stage the expression level at different developmental stages and in different organs, and the sequence similarity to known proteins and genes, MdPIN1 and MdPIN10 analogously to AtPIN1, would be involved in PAT directed to the root apical meristems[31]. On the other hand, MdPIN4 and MdPIN7, similarly to AtPIN3 and AtPIN7, may have an important role in hypocotyl elongation because they are expressed in hypocotyl epidermis and bundle sheath. However, immunolocalization and GFP studies are necessary to further investigate which elements in apple are the physiological orthologs to those of Arabidopsis.

Moreover, a general decrease in the transcript amount of *MdLAX1*, *MdLAX2*, *MdLAX3*, *MdPIN1 *and *MdPIN10 *was observed during senescence and ageing of different organs/tissues such as seed, leaf, flower and cortex. As discussed above, it is well known that a drop in auxin level occurs during leaf senescence preceding abscission. It has also been extensively demonstrated that the majority of Aux/IAA transcripts increase after auxin application in several systems, making them good molecular markers for the auxin endogenous level [18,27,61-63]. Furthermore, analogously to the PIN clones herein isolated, transcripts of some PINs were found to increase following auxin application in poplar, pea and *A. thaliana *[60,64,65]. Therefore, it is conceivable that the change in *MdIAA7 *and *PIN *transcripts occurring at certain developmental stages is at least partially auxin-related. On the other hand, the clone *MdIAA3 *may represent a gene that is highly expressed and only partially controlled by auxin. In addition, a slight increase in *MdLAX1*, *MdPIN4 *and *MdPIN7 *transcripts was monitored in senescing flowers, as reported during daffodil flower senescence [66]. These results indicate that the expression of the PAT elements, besides being under auxin control, is developmentally and tissue specifically regulated, as previously reported for hybrid aspen and *A. thaliana*, respectively [64,65].

### PAT transcripts are differentially regulated during abscission induction

The expression analysis performed along abscission induction pointed out a general increase in the transcription of PAT elements in cortex, whereas a predominant decrease was observed in seeds. Furthermore, considering that *MdPIN1 *and *MdPIN7 *are the most abundant transcripts (Figure 3, Figure 4, and Table 1) and that *MdPIN10 *and *MdPIN4 *were not detected in seed, it appears that during the fruitlet abscission a decrease in PAT at the level of the seed occurs. On the other hand, the low growth rate [61] and the high level of PAT transcripts, such as *MdIAA3 *and *MdIAA7 *(this work) observed in the cortex of AF, indicate a delay in development. This hypothesis is strengthened by the up-regulation of *MdARR6*, *MdARR3 *and *MdARR4 *occurring in the cortex of AF. In fact, the type A ARR is the best indicator of the level of the cytokinin (the hormone that stimulates cell division [67]), in which this phenomenon occurs in fruit exclusively at early development.

The cellular concentration of auxin is due to both transport rate and homeostasis, but in the wood forming tissues, the latter is largely predominant on synthesis, catabolism and conjugation due to the scarce capacity of the cells [64]. The peduncle and the AZ are mainly made up of this type of cells. Different patterns were found in transcripts accumulation: *MdPIN10 *decreased in peduncles and in the AZ, whereas *MdPIN4 *increased in peduncle and decreased in AZ, indicating a different nature of the tissues. We may hypothesize that during abscission induction the decrease in auxin flow coming from the seeds determines a change in the peduncle both in terms of auxin level, as assessed by the *MdIAA7 *transcript accumulation and in terms of PAT, because peduncle is the fruitlet organ in which *MdPIN4 *and *MdPIN10 *are mostly expressed and that the diverse PIN mutants display partially different phenotypes (as previously discussed). The vertical flux may be hampered due to an increase in the apolar distributed efflux carriers. In this scheme, the auxin flow through the AZ will progressively decrease, leading to AZ activation. Nevertheless, auxin at this stage may play a pivotal role in tissue differentiation leading to wood formation [68], as demonstrated by the isolation of auxin related clones putatively involved in the process [69,70]. In this case, the auxin flow may be directed, according to the signal flow canalization hypothesis [71], through a lateral transport mediated by specifically localized AECs such as *MdPIN4*. The difference in development between AF and NAF is further confirmed by the higher level of *MdLAX1 *transcripts and by the reduced diameter of the lateral fruitlet peduncle. In agreement to what was observed for the hypocotyls during de-etiolation, the differential expression may also be related to a higher elongation rate. Nevertheless, despite the fact that AF peduncles are longer than NAF, there was no difference in the growth rate, thus indicating that the differential expression is unrelated to cell elongation but mainly related to tissue formation. Concerning AZ, the transient decrease in *MdLAX1 *and the continuous decrease in *MdPIN10 *and *MdPIN4 *transcripts may indicate a redistribution of auxin.

### Abscission induction and dominance

Abscission in apple is proposed to be due to competition between auxin flows coming from the different fruitlets in a dominance-like fashion [37]. The data herein presented indicate that during abscission induction there is a clear decrease in PAT-related transcripts in the seed, the key element in fruitlet dominance. It is then likely that the drop in auxin export capacity of AF leads to a weak auxin flow which cannot compete with the strong flux from the central fruitlet. As previously discussed, dominance is related to the capacity of the apex to attract cytokinins, which are positive regulators of type A ARR expression. Indeed, a decrease in *MdARR6 *and *MdARR3 *transcripts was observed in seeds during abscission induction, thus indicating a shortage in cytokinin. Nevertheless, the low level of cytokinin is likely due to a low biosynthetic rate rather than to a reduced import. It has been shown that there are several IPT genes expressed at specific stages of seed development [72] and there is a specific developmental window during seed formation in which a large increase in seed cytokinin is observed in cereals and beans [73-76]. At this stage, cytokinins are believed to strengthen sink activity and to induce endosperm proliferation. Furthermore, the chalazal endosperm is thought to be involved in importing assimilates into the developing seeds [77]. Since cytokinins in seed are not exported, it is likely that they play a role as local mediators rather than systemic signals [78,79]. Considering MAX2, an additional element involved in apical dominance, its expression pattern in the different tissues is opposite to that of *MdLAX2/3*, *MdARR6 *and *MDPIN10*, especially in seedlings. This divergence may indicate some common regulatory mechanisms in terms of transcription. Nevertheless, such relation is lacking in the tissues undergoing abscission, indicating that MdMAX2 may not play an important function in PIN regulation as reported in other systems but instead act in other processes such as senescence [80,81].

## Conclusion

It appears that the expression level of genes related to auxin and the PAT elements are tightly correlated to abscission induction driven by dominance in apple. It is feasible that the strong auxin flow characterizing the central fruitlet dominates the weak ones [41]. Nevertheless, it is not clear what is the cue determining the fall in the seed PAT that triggers abscission. The best candidate is development, which is also affected by carbohydrate availability [82]. The developmental stage acts through the same auxin flow (small seeds produce less auxin and cytokinins) and with the likely involvement of ethylene. Indeed, ethylene has already been proven to affect auxin transport [83]. In this study, ethylene was also clearly shown to repress transcript accumulation of *MdLAX1*, *MdLAX2*, *MdPIN1*, *MdPIN10*, *MdARR6 *and *MdARR3*, indicating that the increase in its evolution at this stage may modulate seed development, leading to a reduced auxin export and the induction of abscission.

## Methods

### Plant material

The experiments were performed on *Malus × domestica*. Eight-year old apple trees (cv Golden Delicious/M9) were grown interspaced by pollinator trees (cv Stark Red). In order to investigate the expression profile of the identified clones in different tissues and in different physiological conditions, seed and cortex from ripe fruits at harvest, flowers at full bloom and un-pollinated senescing flowers, shoot, leaf and senescing leaf and seed, peduncle and cortex of fruitlets at 7 days after petal fall (APF) were collected. Furthermore, since seedlings are the usual system to study elements involved in PAT, seeds were sown in half MS media in the dark. Five days after germination, seedlings were either left for a further 4 days in the dark or moved to light. Root, cotyledon and hypocotyl were collected from seedlings at 5 days after germination and after 4 days of de-etiolation or darkness.

Fruitlets differing in the abscission potential were obtained as previously described with some minor modifications [39]. Abscising fruitlets (AF) were obtained from lateral fruitlets of clusters borne on trees sprayed with benzyladenine (BA) at 200 μg·L^-1 ^when the average fruit diameter was around 10 mm (15 days APF). The non-abscising fruitlets (NAF) were comprised of central fruitlets of clusters in which all the laterals had been removed 7 days APF. Seed, cortex, peduncle and abscission zone (AZ) were collected from NAF and AF at 0, 3, 5 and 7 days after the BA treatment of the AF population. The northern blot analysis (Additional file 10) was performed as previously described with the *MdACO1 *probe [40,84]. Results confirmed the validity of the populations obtained because the increase in *MdACO1 *transcript amount showed to be a reliable abscission marker [38,40,84]. Peduncle length and diameter of NAF and AF were measured with a calibre at 0, 3, 5, 7, 9 and 11 days after BA treatment. Statistical analysis (T-test) was performed with the excel package. The equation and the regression coefficient were also calculated.

### Hormone treatments

Auxin treatment was performed only on the peduncle, because the peduncle is the tissue most respondent to auxin [61]. IAA was dissolved in a constant amount of alcohol at a final concentration of 0.1, 1, 5 and 10 mM. The mock control consisted of peduncle treated with water and the quantity of alcohol used for auxin solubilisation. The entire experiment was performed in the dark. The central part of the peduncle (1.5 cm) was isolated from lateral fruitlets at 15 days after petal fall (DAPF) (T0), immersed in the auxin solution and a vacuum applied for 10 min, and then was left for an additional 120 min under normal conditions and sampled. Entire apple fruitlet clusters at 15 DAPF were treated with propylene (1000 μL·L^-1^) or 1-MCP (1 μL·L^-1^) or left untreated (control) as described in [61]. Tissue was collected at the beginning of the experiment (T0) and after 24 hours for molecular analysis.

### Clone isolation and expression analysis

Total RNA extraction and single strand cDNA synthesis were performed as previously reported [85,39]. The isolation of the partial clones was performed with degenerate primers designed on conserved areas of the ORFs (Table 2) [86]. The reaction was performed in 1× PCR Buffer (Amersham Pharmacia), 0.25 mM dNTPs, 1 μM primer forward, 1 μM primer reverse, and 0.05 U/μl of Taq (Amersham Pharmacia). The PCR profile was as follows: 5 min hold at 95°C and 40 cycles composed by 60 s at 94°C, 60 s at the primer annealing temperature (Tm) and 90 s of extension at 72°C. Tm was as reported in Table 1. The cycles were followed by a final step of 7 min at 72°C.

**Table 2:** List of degenerate primers utilized in the isolation of genes

Clone		Primers		L	Tm
		**forward**	**reverse**	**bp**	**°C**

LAX	S	5'-AACCAYGTYATHCAGTGGTT-3'	5'-GGRATGAYRTARACDGTGAA-3'	840	58
	
	M	NHVIQWF	SFTVYIIP		

PIN	S	5'-CARWGYATHTGGTAYAC-3'	5'-GCAYYARCATYCCAAADAT-3'	1400	57
	
	M	QCIIWY	VIFGMLM		

ARR type	S	5'-CATGTTYTNGCBGTTGATG-3'	5'-CATNCCRGGCATDSAGTARTC-3'	200	60
	
A	M	HVLAVDDS	TDYSMPGM		

Degenerate primers used for the isolation of partial clones of LAX, PIN, and ARR type A identified in this study. S indicates the oligonucleotide sequence while M the conserved protein sequence chosen to design the primers. The amplification length of the product (L) expressed in bp and the annealing temperature are also reported.

The 3'UTR of the clones was subsequently obtained with specific primers (Table 3) as previously described [40]. Briefly the 3'RACE was performed with a gene specific forward primer and the anchored oligo dT as the reverse primer. Annealing temperature was as reported in Table 2.

**Table 3:** List of primers utilized in the isolation of the 3' part of the genes isolated in this study

Clone	Primers	Tm
LAX1	5'-TTTCTCTCTCCTCCCCAAGAAC-3'	68

LAX2/3	5'-CTTTGGATCTGTCATCCAACTTATAG-3'	71

PIN1/10	5'-GGCCTCACTTGGTCTCTAGTCTCA-3'	70

PIN4/7	5'-GTATTCGAGCCTCATTGGTCTCATC-3'	70

ARR3/4	5'-CGAGGGATGTCGGAGTGGAAGAG-3'	69

ARR6	5'-CAGCGGGACGAGAGCTCTGGAG-3'	69

Specific primers used in the 3' RACE for the isolation of the 3' part of LAX, PIN, and ARR type A. The annealing temperature is also reported.

The genomic sequences of *MdPIN1 *and *MdPIN10 *were obtained from the amplification of DNA extracted from leaves of Golden Delicious apples with the Qiagen DNA easy according to manufacturer instructions. The template (100 ng) was amplified in 1× PCR buffer (Amersham), 0.03 U μL^-1 ^Taq (Amersham Pharmacia), 0.3 μM of primer forward (5'-CGGGATCCATTGTCTCCA-3') and 0.3 μM of primer reverse (5'-GGAAACTCCATTGCAGCT-3'). The amplification profile was as follows: an initial denaturation for 5 min at 95°C, 40 cycles composed by 60 s at 94°C, 90 s at 62°C and 90 s at 72°C, and a final step of 7 min at 72°C

Amplification fragments were electrophoresed in agarose gel, purified and cloned into pGEM-T Easy vector (Promega). Positive clones were grown and plasmids were isolated with the Miniprep Kit (Qiagen) and sequenced using the ABIPRISM BigDye Terminator v3.1 kit (Applied Biosystems).

Contigs were assembled by the SeqMan software (DNAstar package) while sequence comparisons were performed using BlastX and BlastN algorithms (NCBI, National Centre for Biotechnology Information). Sequence alignment between the deduced proteins of the clones identified here and those from *LAX *and *PIN *members of *Arabidopsis thaliana *(*AtAUX1*, NP_565882; *AtLAX1*, NP_195744, NP_974719; *AtLAX2*, NP_179701; *AtLAX3*, NP_177892; *AtPIN1*, NP_177500, AF089085; *AtPIN2*, NP_568848; *AtPIN3*, NP_177250; *AtPIN4*, NP_565261, NP_849923; *AtPIN5*, NP_197157; *AtPIN6*, NP_177836; *AtPIN7*, NP_564189, NP_849700, NP_001077584; *AtPIN8*, NP_197014), *Medicago truncatula *(*MtLAX1*, Q9FEL8; *MtLAX2*, Q9FEL7; *MtLAX3*, Q9FEL6; *MtLAX4*, Q8L884; *MtLAX5*, Q8L883; *MtPIN1*, AAM55297; *MtPIN2*, AAM55298; *MtPIN3*, AAM55299; *MtPIN4*, AAM55300; *MtPIN5*, AAM55301; *MtPIN6*, AAT48627; *MtPIN7*, AAT48628; *MtPIN8*, DAA05219; *MtPIN9*, AAT48629; *MtPIN10*, AAT48630) and *Populus tremula × Populus tremuloides *(*PttLAX1*, AAF21982; *PttLAX2*, AAG17171; PttLAX3, AAK58522; *PttPIN1*, AAG17172; *PttPIN2*, AAM54033; *PttPIN3*, AAM54034) were performed with the aid of the Clustal W algorithm using default settings (MegAlign software, DNAstar package).

Expression analysis of the clones was performed by the semi-quantitative PCR implemented with ^33^P labeled primers as previously described [40]. Specific conditions of this experiment are reported in Table 3. Data concerning the abscission experiment are expressed as fold induction of the ratio between the level of expression in AF and NAF populations, corrected for the transcript amount of the constitutive gene (*MdUBI*). Data referring to auxin application are presented as fold induction after correction for the level of the constitutive gene (*MdUBI*). Changes of at least 0.5 fold magnitudes are considered significant.

## Abbreviations

AF: Abscising Fruitlet; ARR: Arabidopsis Response Regulator; *At*: *Arabidopsis thaliana*; Aux/IAA: Aux/IAA; AUX: AUXin; AZ: abscission zone; DAPF: days after petal fall; *dad*: Decreased Apical Dominance; GFP: green fluorescent protein; IAA: indol acetic acid; LAX: Like AUX; MAX: Maximum Axillary; *Md*: *Malus × domestica*; *Mt*: *Medicago truncatula*; NAF: Non Abscising Fruitlet; PAT: Polar Auxin Transport; PIN: PIN FORMED; *rms*: ramosus; T0: time 0; UBI: UBIquitin.

## Authors' contributions

VDC conceived and designed the experimental study, performed the experiments, and wrote the manuscript. RV gave intellectual guidance and financially supported the research. AR conceived and designed the experimental study, gave intellectual guidance, financially supported the research and wrote the manuscript. All authors read and approved the final manuscript.

## Supplementary Material

Additional file 1 **Scheme of the PINs isolated in this study**. The scheme illustrates the partial clones of PIN isolated in this study. Rectangles represent the CDS whereas the line indicates the UTR. The blank rectangle and the dotted line represent the missing sequence. The stop codon is reported in capital letter between the CDS and the 3' UTR. The string of four A, where present, indicates the polyA tail. The length of the parts is reported in base pairs (bp) at the bottom. Click here for file

Additional file 2 **Scheme of the LAX isolated in this study**. The scheme illustrates the partial clones of LAX isolated in this study. Rectangles represent the CDS whereas the lines indicate the UTR. The blank rectangle and the dotted line represent the missing sequence. The stop codon is reported in capital letter between the CDS and the 3' UTR. The string of four A, where present, indicates the polyA. The length of the parts is reported in base pairs (bp) at the bottom. Click here for file

Additional file 3 **Identity values among the PIN isolated in this study with those of several species**. Identity values among the protein sequences of PIN isolated in this study from *Malus × domestica *(*Md*) and those of *Arabidopsis thaliana *(At), *Medicago truncatula *(Mt), *and Populus tremula × Populus tremuloides *(Ptt). (*MdPIN1_A*, EF406255; *MdPIN1_B*, EF406256; *MdPIN4*, EF406257; *MdPIN7_A*, EF406258; *MdPIN7_B*, EF406259; *MdPIN10_A*, EF406260; *MdPIN10_B*, EF406261; *AtPIN1*, NP_177500; AF089085; *AtPIN2*, NP_568848; *AtPIN3*, NP_177250; *AtPIN4*, NP_565261, NP_849923; *AtPIN5*, NP_197157; *AtPIN6*, NP_177836; *AtPIN7*, NP_564189, NP_849700, NP_001077584; *AtPIN8*, NP_197014; *MtPIN1*, AAM55297; *MtPIN2*, AAM55298; *MtPIN3*, AAM55299; *MtPIN4*, AAM55300; *MtPIN5*, AAM55301; *MtPIN6*, AAT48627; *MtPIN7*, AAT48628; *MtPIN8*, DAA05219; *MtPIN9*, AAT48629; *MtPIN10*, AAT48630; *PttPIN1*, AAG17172; *PttPIN2*, AAM54033; *PttPIN3*, AAM54034). Click here for file

Additional file 4 **Identity values of the intronic regions**. The values are the identity percentages of the intronic nucleotide sequences of *MdPIN1 *(EF406268), *MdPIN10_A *(EF406269) and *MdPIN10_B *(EF406270) obtained with the clastalW alignment. Length is represented on the left and expressed as base pairs (bp). Click here for file

Additional file 5 **Identity values among the LAX isolated in this study and those of several species**. Identity values among the protein sequencesLAX isolated in this study from *Malus × domestica *(Md) with those of *Arabidopsis thaliana *(At), *Medicago truncatula *(Mt), *and Populus tremula × Populus tremuloides *(Ptt). (*MdLAX1*, EF406263; *MdLAX2*, EF406264; *MdLAX3*, EF406262; *AtAUX1*, NP_565882; *AtLAX1*, NP_195744, NP_974719; *AtLAX2*, NP_179701; *AtLAX3*, NP_177892; *MtLAX1*, Q9FEL8; *MtLAX2*, Q9FEL7; *MtLAX3*, Q9FEL6; *MtLAX4*, Q8L884; *MtLAX5*, Q8L883; *PttLAX1*, AAF21982; *PttLAX2*, AAG17171; PttLAX3, AAK58522). Click here for file

Additional file 6 **Expression analysis with degenerative primers**. The expression analysis was performed with PIN degenerate primers on cDNA of peduncle. Click here for file

Additional file 7 **Expression analysis of PINs following auxin application**. The expression analysis of PIN was preformed on CDNA from peduncle tissue treated with auxin at different concentration: 0.1, 1, 5, 10 mM and the mock control (M) for 90 min. Expression data are corrected for the constitutive gene and presented as fold induction compared to the beginning of the experiment (T0). Click here for file

Additional file 8 **PIN Phylogenetic tree**. Phylogenetic tree of the PINs isolated in this study from *MalusXdomestica (Md) *and those of *Arabidopsis thaliana *(At), *Medicago truncatula *(Mt), *Oryza sativa *(Os)*cultivar indica (ind) *and *japonica (jap)*, *Populus tremula × Populus tremuloides *(Ptt), *Populus balsamifera subsp. trichocarpa *(Ptric), *Physcomitrella patens subsp patens *(Pp) and several varieties of *Vitis vinifera *(Vv) from different varieties. Bootstrap values are indicated. Var means transcript variation. The accession number is reported at the end of the sequence name. Click here for file

Additional file 9 **LAX phylogenetic tree**. Phylogenetic tree of the LAX isolated in this study from *MalusXdomestica (Md) *and those of *Arabidopsis thaliana *(At), *Medicago truncatula *(Mt), *Oryza sativa *(Os)*cultivar indica (ind) *and *japonica (jap)*, *Populus tremula × Populus tremuloides *(Ptt), *Populus balsamifera subsp. trichocarpa *(Ptric), *Physcomitrella patens subsp patens *(Pp) and several varieties of *Vitis vinifera *(Vv) from different varieties. Bootstrap values are indicated. The accession number is reported after the sequence. The accession number is reported at the end of the sequence name. Click here for file

Additional file 10 ***MdACO1 *expression analysis during abscission**. The expression analysis was performed by northern blot on the sample utilized in this study: AF (abscising fruitlets) and NAF (non-abscising fruitlets) at 0, 3 5 and 7 days during abscission induction. The control is represented by 18S. Click here for file
