# Supplementary figures and images for: Dominance induction of fruitlet shedding in Malus × domestica (L. Borkh): molecular changes associated with polar auxin transport
Source: BMC Plant Biol. 2009 Nov 26;9:139. doi: 10.1186/1471-2229-9-139 (PMC2809502; doi:10.1186/1471-2229-9-139)

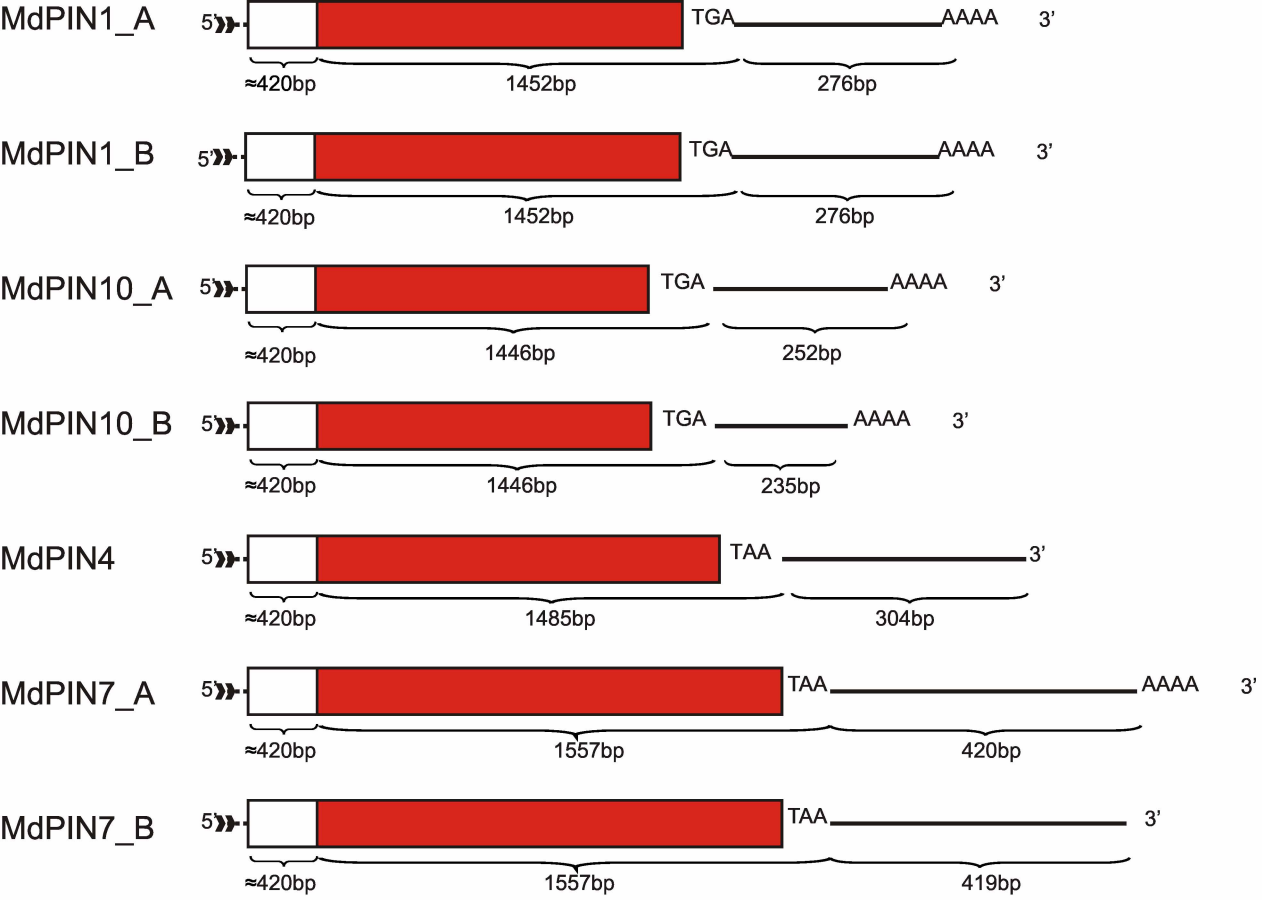

Supplement: Additional file 1 — Scheme of the PINs isolated in this study. The scheme illustrates the partial clones of PIN isolated in this study. Rectangles represent the CDS whereas the line indicates the UTR. The blank rectangle and the dotted line represent the missing sequence. The stop codon is reported in capital letter between the CDS and the 3' UTR. The string of four A, where present, indicates the polyA tail. The length of the parts is reported in base pairs (bp) at the bottom. [file 1471-2229-9-139-S1.pdf]

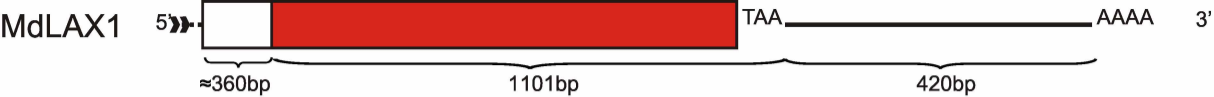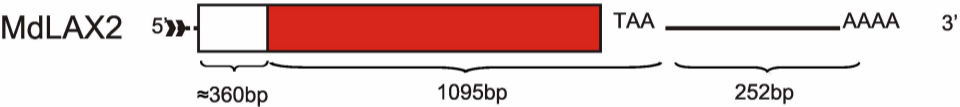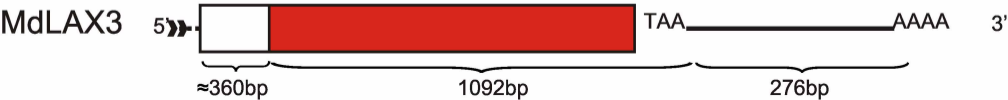

Supplement: Additional file 2 — Scheme of the LAX isolated in this study. The scheme illustrates the partial clones of LAX isolated in this study. Rectangles represent the CDS whereas the lines indicate the UTR. The blank rectangle and the dotted line represent the missing sequence. The stop codon is reported in capital letter between the CDS and the 3' UTR. The string of four A, where present, indicates the polyA. The length of the parts is reported in base pairs (bp) at the bottom. [file 1471-2229-9-139-S2.pdf]

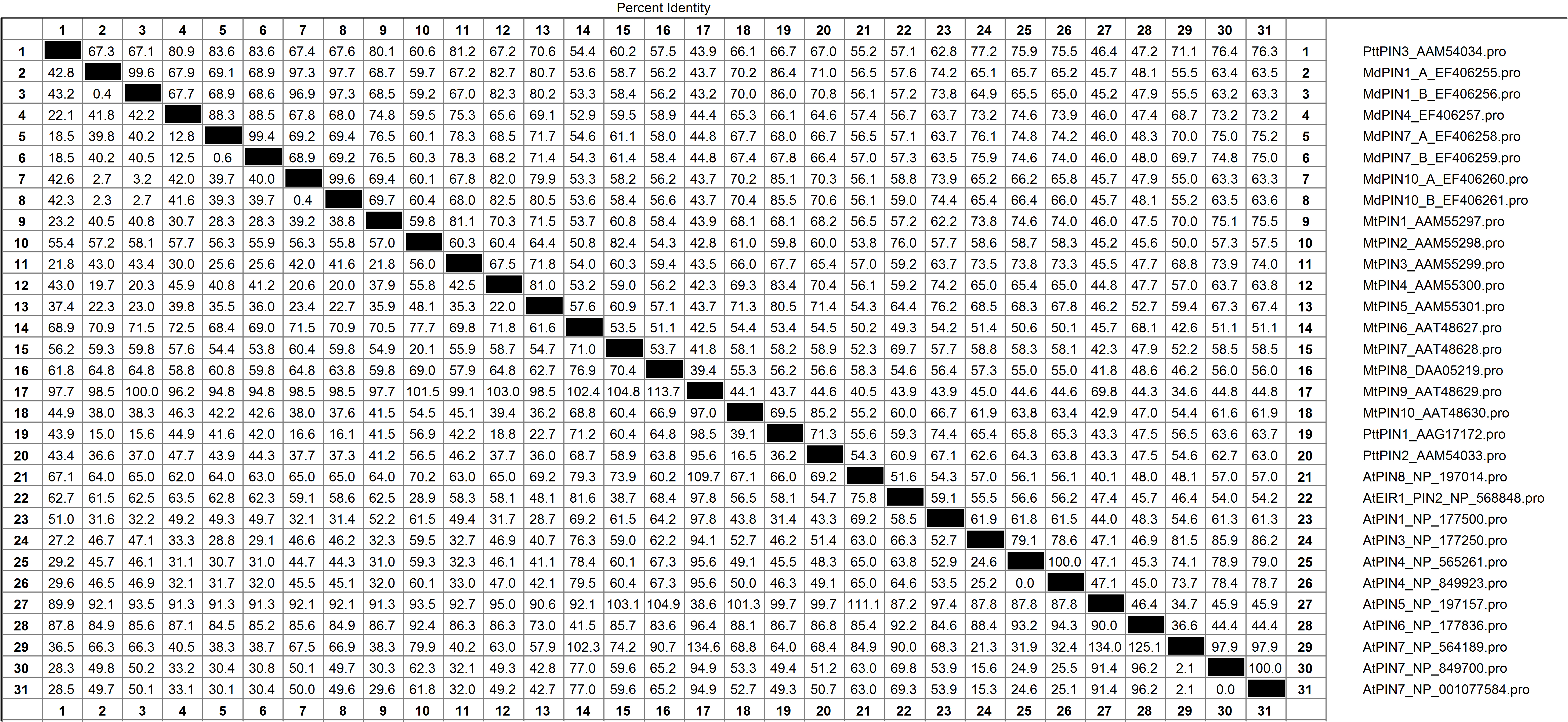

Supplement: Additional file 3 — Identity values among the PIN isolated in this study with those of several species. Identity values among the protein sequences of PIN isolated in this study from Malus × domestica (Md) and those of Arabidopsis thaliana (At), Medicago truncatula (Mt), and Populus tremula × Populus tremuloides (Ptt). (MdPIN1_A, EF406255; MdPIN1_B, EF406256; MdPIN4, EF406257; MdPIN7_A, EF406258; MdPIN7_B, EF406259; MdPIN10_A, EF406260; MdPIN10_B, EF406261; AtPIN1, NP_177500; AF089085; AtPIN2, NP_568848; AtPIN3, NP_177250; AtPIN4, NP_565261, NP_849923; AtPIN5, NP_197157; AtPIN6, NP_177836; AtPIN7, NP_564189, NP_849700, NP_001077584; AtPIN8, NP_197014; MtPIN1, AAM55297; MtPIN2, AAM55298; MtPIN3, AAM55299; MtPIN4, AAM55300; MtPIN5, AAM55301; MtPIN6, AAT48627; MtPIN7, AAT48628; MtPIN8, DAA05219; MtPIN9, AAT48629; MtPIN10, AAT48630; PttPIN1, AAG17172; PttPIN2, AAM54033; PttPIN3, AAM54034). [file 1471-2229-9-139-S3.jpeg]

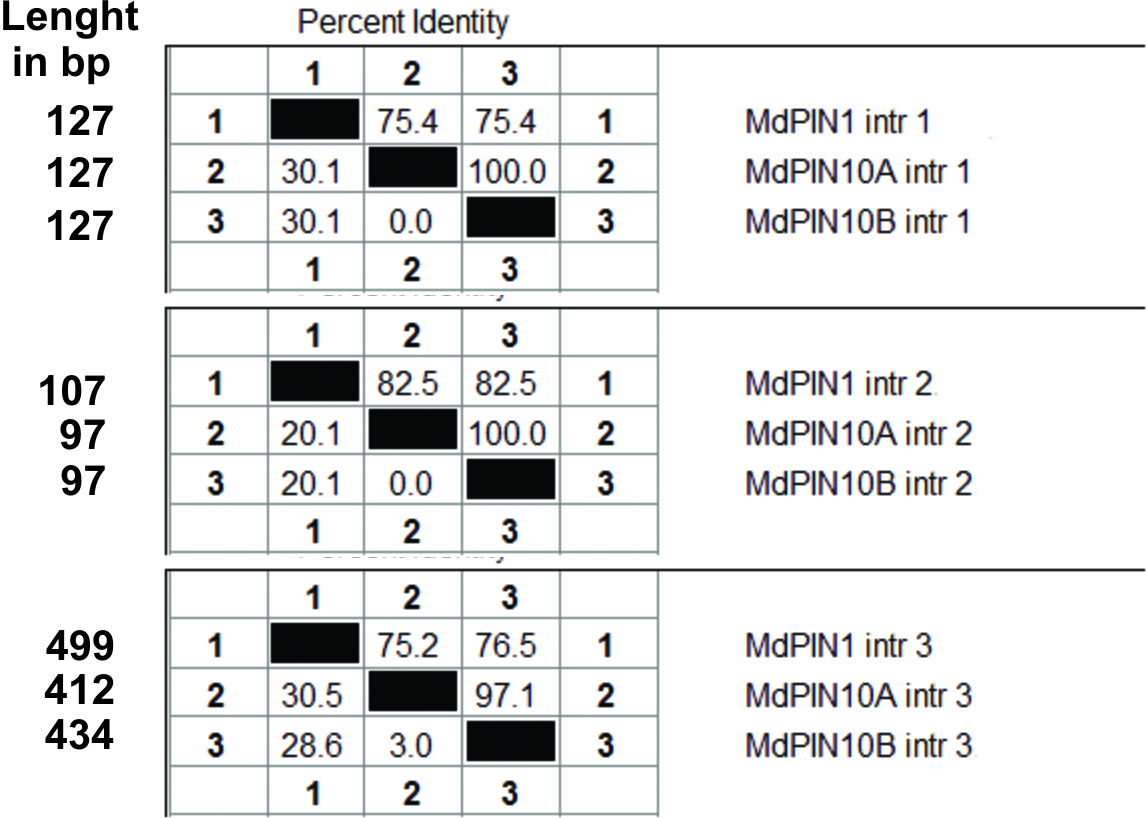

Supplement: Additional file 4 — Identity values of the intronic regions. The values are the identity percentages of the intronic nucleotide sequences of MdPIN1 (EF406268), MdPIN10_A (EF406269) and MdPIN10_B (EF406270) obtained with the clastalW alignment. Length is represented on the left and expressed as base pairs (bp). [file 1471-2229-9-139-S4.tiff]

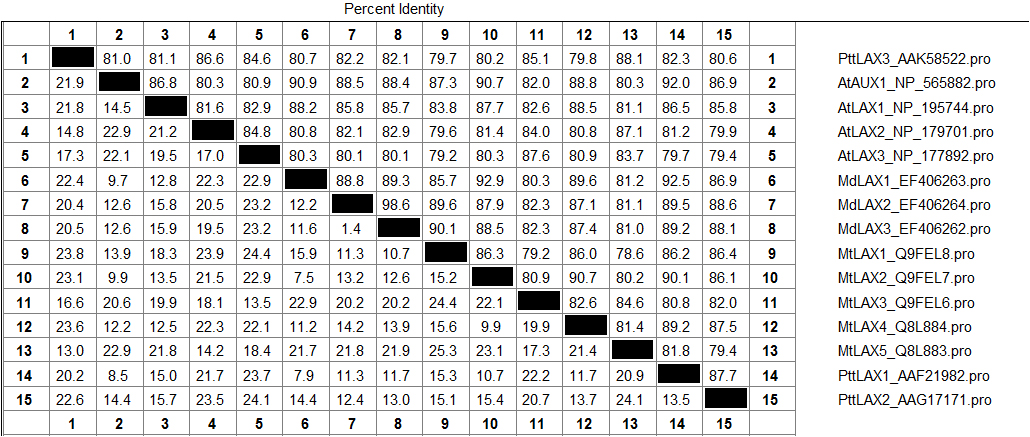

Supplement: Additional file 5 — Identity values among the LAX isolated in this study and those of several species. Identity values among the protein sequencesLAX isolated in this study from Malus × domestica (Md) with those of Arabidopsis thaliana (At), Medicago truncatula (Mt), and Populus tremula × Populus tremuloides (Ptt). (MdLAX1, EF406263; MdLAX2, EF406264; MdLAX3, EF406262; AtAUX1, NP_565882; AtLAX1, NP_195744, NP_974719; AtLAX2, NP_179701; AtLAX3, NP_177892; MtLAX1, Q9FEL8; MtLAX2, Q9FEL7; MtLAX3, Q9FEL6; MtLAX4, Q8L884; MtLAX5, Q8L883; PttLAX1, AAF21982; PttLAX2, AAG17171; PttLAX3, AAK58522). [file 1471-2229-9-139-S5.jpeg]

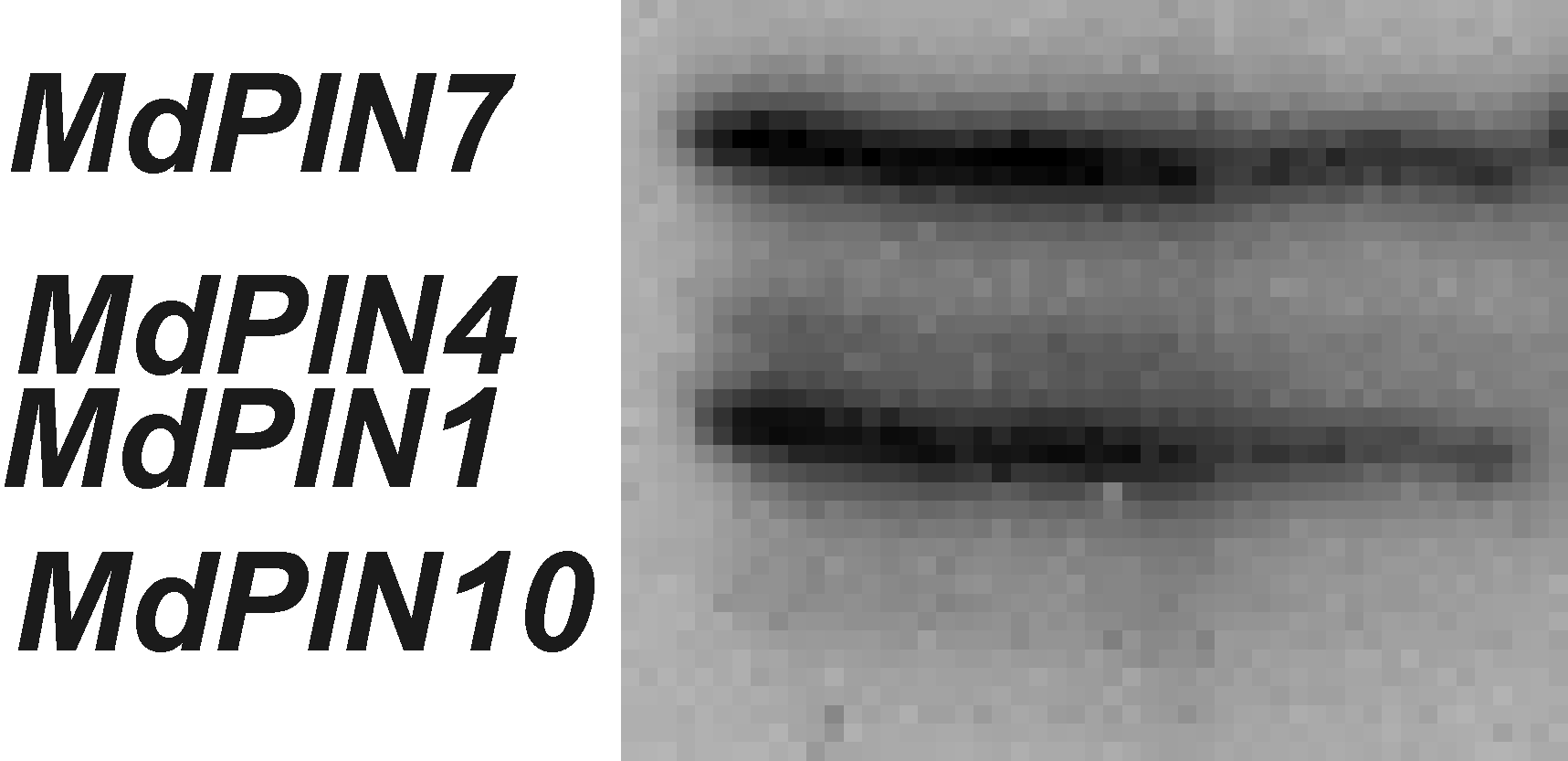

Supplement: Additional file 6 — Expression analysis with degenerative primers. The expression analysis was performed with PIN degenerate primers on cDNA of peduncle. [file 1471-2229-9-139-S6.tiff]

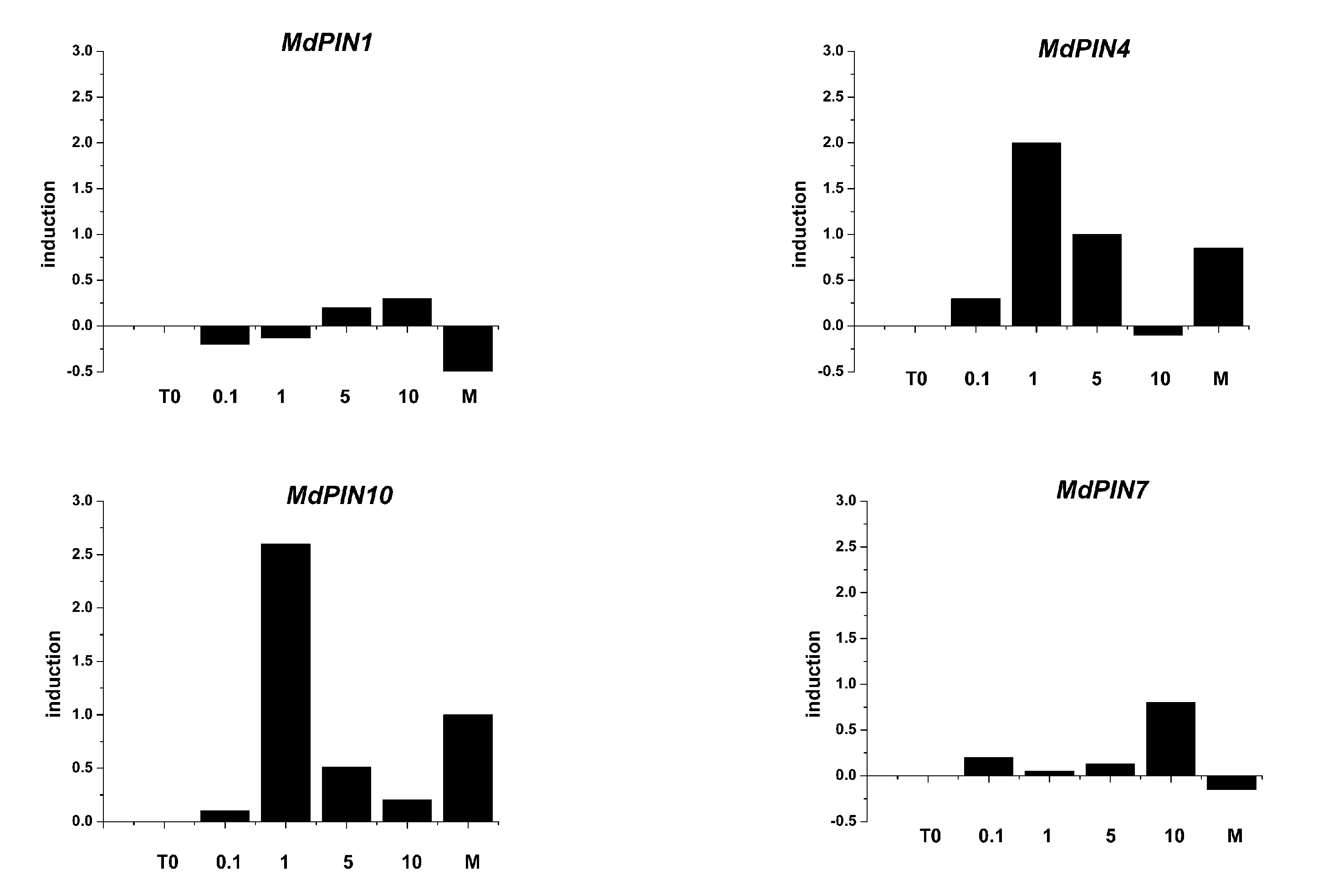

Supplement: Additional file 7 — Expression analysis of PINs following auxin application. The expression analysis of PIN was preformed on CDNA from peduncle tissue treated with auxin at different concentration: 0.1, 1, 5, 10 mM and the mock control (M) for 90 min. Expression data are corrected for the constitutive gene and presented as fold induction compared to the beginning of the experiment (T0). [file 1471-2229-9-139-S7.jpeg]

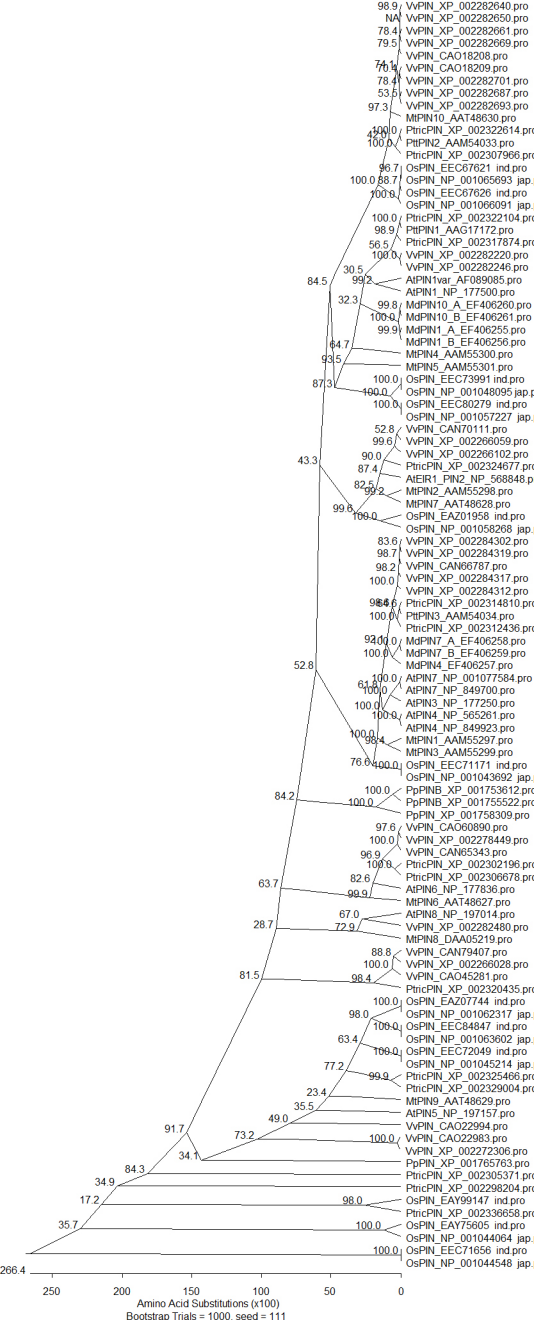

Supplement: Additional file 8 — PIN Phylogenetic tree. Phylogenetic tree of the PINs isolated in this study from MalusXdomestica (Md) and those of Arabidopsis thaliana (At), Medicago truncatula (Mt), Oryza sativa (Os)cultivar indica (ind) and japonica (jap), Populus tremula × Populus tremuloides (Ptt), Populus balsamifera subsp. trichocarpa (Ptric), Physcomitrella patens subsp patens (Pp) and several varieties of Vitis vinifera (Vv) from different varieties. Bootstrap values are indicated. Var means transcript variation. The accession number is reported at the end of the sequence name. [file 1471-2229-9-139-S8.pdf]

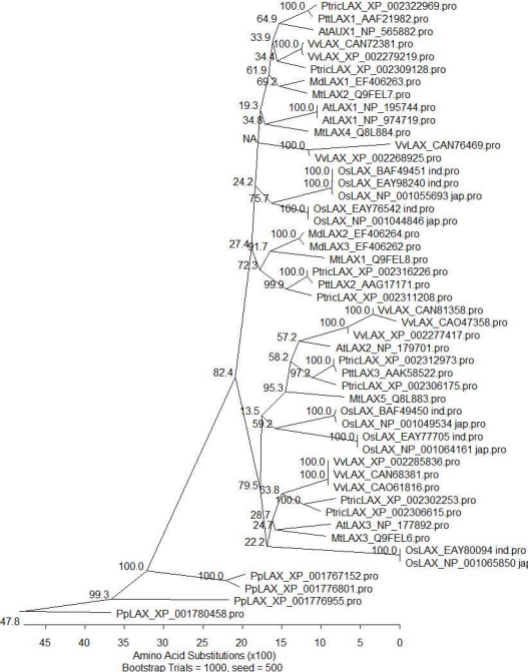

Supplement: Additional file 9 — LAX phylogenetic tree. Phylogenetic tree of the LAX isolated in this study from MalusXdomestica (Md) and those of Arabidopsis thaliana (At), Medicago truncatula (Mt), Oryza sativa (Os)cultivar indica (ind) and japonica (jap), Populus tremula × Populus tremuloides (Ptt), Populus balsamifera subsp. trichocarpa (Ptric), Physcomitrella patens subsp patens (Pp) and several varieties of Vitis vinifera (Vv) from different varieties. Bootstrap values are indicated. The accession number is reported after the sequence. The accession number is reported at the end of the sequence name. [file 1471-2229-9-139-S9.pdf]

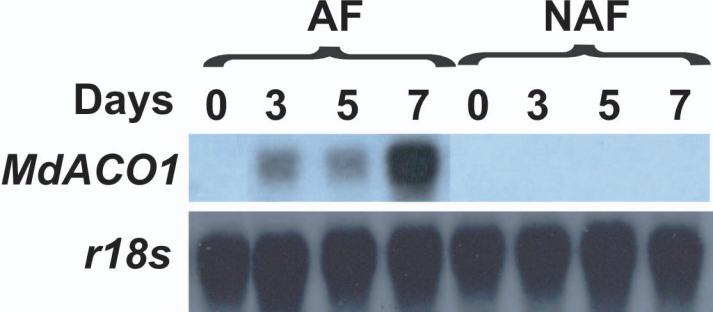

Supplement: Additional file 10 — MdACO1 expression analysis during abscission. The expression analysis was performed by northern blot on the sample utilized in this study: AF (abscising fruitlets) and NAF (non-abscising fruitlets) at 0, 3 5 and 7 days during abscission induction. The control is represented by 18S. [file 1471-2229-9-139-S10.pdf]
